# The effects of habitat type and pathogen infection on tick host-seeking behaviour

**DOI:** 10.1017/S0031182021001554

**Published:** 2022-01

**Authors:** Elise A. Richardson, Caitlin E. Taylor, Brittney Jabot, Estelle Martin, Carl N. Keiser

**Affiliations:** 1 Department of Biology, University of Florida, Gainesville, FL 32611, USA; 2 Department of Entomology and Nematology, University of Florida, Gainesville, FL 32611, USA

**Keywords:** Habitat, lone star tick, questing behaviour, *Rickettsia*, tick-borne disease

## Abstract

Tick-borne pathogens pose a significant risk to livestock, wildlife and public health. Host-seeking behaviours may depend on a combination of infection status and environmental factors. Here, we assessed the effects of habitat type and pathogen infection on host-seeking behaviour (questing) in the lone star tick, *Amblyomma americanum*. Ticks were collected using a tick drag from two different habitat types: xeric hammock and successional hardwood forests. Using a standardized assay, we recorded the likelihood of questing for each tick, the average height quested and total time spent questing and then tested each tick for the presence of *Rickettsia* spp. and *Ehrlichia* spp. using conventional polymerase chain reaction. We did not detect *Ehrlichia* in any ticks, although 30% tested positive for *Rickettsia amblyommatis*, a member of the *Rickettsia* spotted fever group. Ticks infected with *R. amblyommatis* spent less time questing compared to uninfected ticks, with infected ticks spending 85 s on average questing and uninfected ticks spending 112 s. Additionally, ticks collected from xeric hammock habitats spent over twice as long questing compared to ticks from successional hardwood forests. Ticks from xeric hammock spent 151 s on average questing while ticks from successional hardwood forest spent only 58 s during a 10-min observation period. These results demonstrate that habitat type and infection status can influence tick host-seeking behaviours, which can play a pivotal role in disease dynamics.

## Graphical abstract

**Figure:**
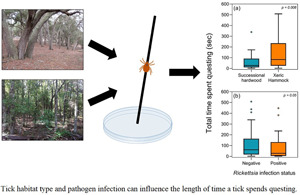

## Introduction

Studying the host-seeking behaviours of disease vectors can provide pivotal information for not only understanding fundamentals of host–parasite biology, but also in designing effective public health interventions. Ticks are responsible for transmitting a greater variety of pathogens than any other arthropod group (Jongejan and Uilenberg, 2004), are the chief source of vector-borne diseases in wildlife and domestic animals and are second only to mosquitoes in transmitting human diseases (De La Fuente *et al*., 2008). As emerging tick-borne diseases are discovered, and as ticks expand their ranges, it is increasingly important to study tick behaviour in response to pathogen infection. Considering their wide host range which apart from domestic animals and humans, it also includes several wildlife species that present an important role in the spreading of ticks and tick-borne pathogens (Bezerra-Santos *et al*., 2021*b*). For example, the lone star tick, *Amblyomma americanum*, is an aggressive human-biting tick that is a competent vector of many pathogens that pose a known threat to public health, including Rocky Mountain Spotted Fever (RMSF), human monocytic ehrlichiosis, tularaemia and heartland virus (Childs and Paddock, 2003). *Amblyomma americanum* is predicted to expand its geographic range northwards from the South and Central United States with changing climatic conditions (Sagurova *et al*., 2019). Given the widening distribution and vectorial capacity of *A. americanum*, it is more critical than ever to study host-seeking behaviour across different habitats.

*Amblyomma americanum* is found in a large variety of habitat types, based mainly on the presence and movement of vertebrate hosts (Koch and Burg, 2006; Willis *et al*., 2012). Larger populations of *A. americanum* are often found in canopied, shaded areas which have an overall wooded habitat providing abundant leaf litter (Semtner and Hair, 1973; Koch, 1984; Koch and Burg, 2006) potentially to reduce water loss while engaging in host seeking in shaded areas (Hair *et al*., 1975; Jaworski *et al*., 1984; Koch, 1984; Needham and Teel, 1991). Tick host-seeking behaviour is called ‘questing’ and refers to a stereotyped series of behaviours where ticks climb tall grasses and other vegetation, halt and extend their forelegs and wait to attach to a passing host (Holderman and Kaufman, 2013; McClure and Diuk-Wasser, 2019). The duration and height at which ticks quest can influence host selection (Randolph and Storey, 1999; Randolph, 2004) but continued questing at greater heights can pose a desiccation risk (Needham and Teel, 1991; Portugal *et al*., 2020). In Texas, *Amblyomma mixtum* ticks quested at greater heights in experimental chambers when relative humidity was held at 95% as compared to 56% (Beck and Orozco, 2015). However, Schulze *et al*. found the opposite pattern in *A. americanum* ticks in New Jersey, where ticks spent the most time questing when temperatures were high and humidity was low (Schulze *et al*., 2001). In contrast to both of these studies, Lane *et al*. did not find any consistent pattern of activity with nymphal *Ixodes pacificus* in regards to temperature, humidity or time of day (Lane *et al*., 2007). In addition, prescribed burns have been suggested as a mitigation strategy for ticks, so we also sought to test whether tick behaviour differs between habitats with varying burn history (Davidson *et al*., 1994; Willis *et al*., 2012; Gleim *et al*., 2014). Although extrinsic factors of the environment play a large role in tick-questing behaviour, intrinsic factors such as pathogen infection also influence host-seeking behaviour (Benelli, 2020). However, few studies address the joint effects of habitat type and infection status on questing behaviour in *A. americanum*.

A major avenue of research in disease vector ecology focuses on how infection status alters vector behaviour and life history traits and, in turn, affects disease risk for hosts. For example, *I*xodes *ricinus* ticks infected with *Borrelia* spp., the causative agent of Lyme disease, exhibit increased lifespan and resistance to desiccation, and as a result have been shown to spend longer periods of time questing compared to uninfected ticks (Herrmann and Gern, 2015). Similarly, *Ixodes scapularis* nymphs infected with *Borrelia burgdorferi* quest at greater heights and show an increased tendency to overcome physical barriers or obstacles in order to quest (Lefcort and Durden, 1996). Busby *et al*. found that ticks infected with *Anaplasma phagocytophilum* showed increased questing speeds and reduced desiccation risk (Busby *et al*., 2012). Since Lyme disease has been the focus of tick research for so long, most of the literature on infection status and tick-questing behaviour has focused on *Ixodes* ticks (Benelli, 2020). However, public health officials are becoming increasingly concerned about tick-borne diseases transmitted by *A. americanum*, and no study has yet focused on infection status and questing behaviour in *A. americanum*.

*Rickettsia amblyommatis* is very prevalent in some *A. americanum* populations with one study finding up to 84% prevalence (Mixson *et al*., 2006). In a study conducted across Florida, *R. amblyommatis* prevalence was found to be between 20 and 50% depending on the time of year, along with other tick-borne pathogens with low prevalence (De Jesus *et al*., 2019). In addition, this pathogen has been detected in other *Amblyomma* spp. species collected from wildlife and domestic animals (Costa *et al*., 2017; Bezerra-Santos *et al*., 2021*a*; Mendoza-Roldan *et al*., 2021). This study aimed to address the joint effects of habitat type and tick-borne pathogen infection (i.e., *Ehrlichia* spp. and *R. amblyommatis*) on *A. americanum* questing behaviour.

## Materials and methods

### Tick collection and maintenance

**Fig. 1.**
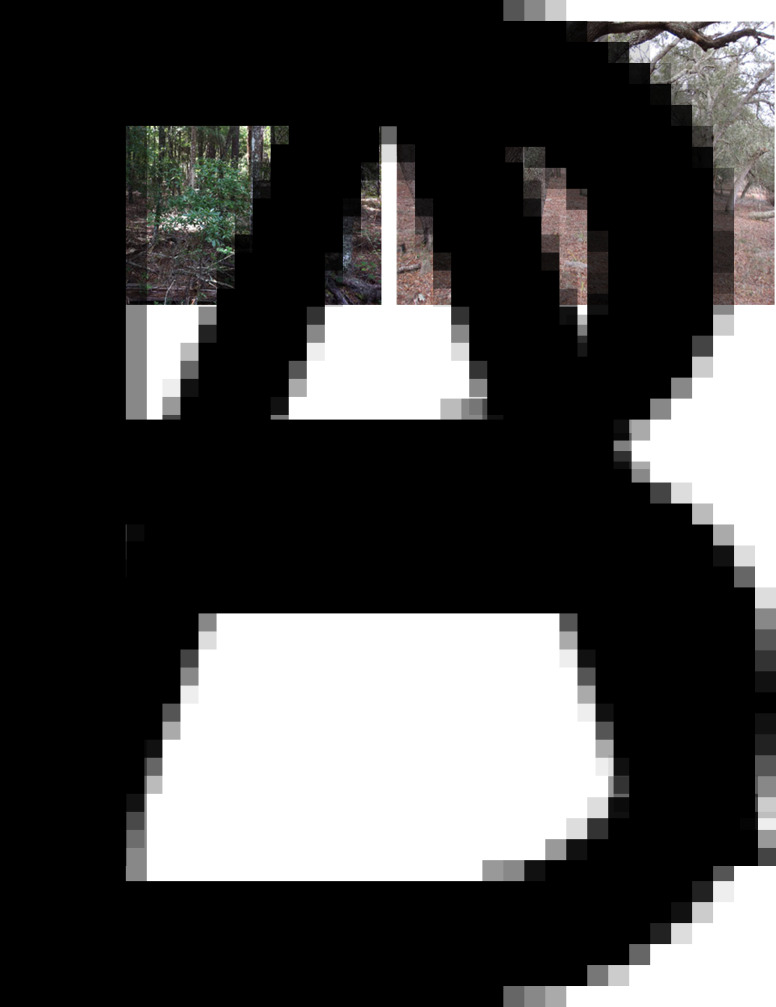
Habitat types where ticks were collected in OSBS: (A) successional hardwood and (B) xeric hammock. Photo credits: Ordway-Swisher Biological Station.

In total, 182 adult and nymphal *A. americanum* ticks were collected in the Ordway-Swisher Biological Station (OSBS) in Hawthorne, Florida, a research and extension facility of the University of Florida. OSBS contains an array of natural and altered habitat types, defined by the Florida Natural Areas Inventory classification system (Guide to the natural communities of Florida: 2010 edition, 2010). Furthermore, OSBS has a detailed history of prescribed fires across each habitat type. Ticks were collected in three habitat types: abandoned fields/pastures, successional hardwood forests and xeric hammocks. Due to the low number of ticks (*n* = 5) collected in the abandoned field/pasture habitat type, only the other two habitat types were considered in the analysis. One additional tick was removed from the analysis as it had a low DNA output after extraction and could not be sequenced for pathogens. Successional hardwood forests are altered landcover characterized by a closed canopy dominated by fast-growing hardwoods, a dense subcanopy and shrub layer (Guide to the natural communities of Florida: 2010 edition, 2010; Fig. 1a). Xeric hammocks are evergreen forests characterized by well-drained sandy soils, a low canopy of various oak trees and an open or shrubby understory (Guide to the natural communities of Florida: 2010 edition, 2010; Fig. 1b). A total of 82 ticks were collected from the xeric hammock habitat types and 95 from the successional hardwoods.

To assess the effects of burn history, two sites were chosen per habitat type, one that had a prescribed burn within the last 4 years and one that had been burned more than 4 years ago. At each collection event, temperature, relative humidity and wind speed were collected using a Kestrel^®^ 3000 Wind Meter (Table S1). Ticks were collected in the late morning to early afternoon to maximize the number of *A. americanum* ticks collected (Schulze *et al*., 2001). Two collection events took place at each habitat type between July and August 2020 using a tick drag, a white canvas sheet that is dragged behind the researcher. Approximately 50 drags were completed at each time of collection and drags were checked for ticks approximately every 30 m. Ticks were placed in glass vials with mesh tops to ensure air flow and brought back to a standardized location for questing assays.

### Questing assays

Questing assays were conducted outdoors on a screened-in porch in a standardized setting exposed to ambient environmental conditions. Questing assays all took place in the same location to reduce the effects of various environmental conditions, as well as to have a consistent location all individual ticks would be tested in. Ticks were tested approximately within 1–3 h after being collected. Questing assays were conducted in a clear plastic plant saucer (12 cm height; 18.5 cm diameter) with a 55 cm wooden dowel with 1 cm demarcations in the middle of the saucer held in place with crafting putty (following methods in Tietjen *et al*., 2020; Supplementary materials). The vial that held the tick was opened and human breath was used to activate/stimulate the tick (Vassallo and Pérez-eid, 2002). The open vial was placed adjacent to the putty so that the tick could leave the vial on its own, walking onto the base of the dowel. The 10 min period began immediately upon the tick leaving the vial. Once the tick reached the dowel, the height at which each quest occurred was recorded and the total amount of time spent questing was quantified using an additional stopwatch. Questing assays were conducted for 10 min total, and questing was defined as a tick-halting motion and extending its forelegs off the dowel. Ticks were classified as non-questing if they never, halted and extended their forearms while on the dowel, this includes if a tick climbs the dowel but does not stop to extend their forearms. For example, if a tick engaged in three questing bouts during the 10-min period, each for 10 s, they were recorded as a total questing time of 30 s and the average height of the three questing bouts was recorded. Following the assay, ticks were placed in a microcentrifuge tube and frozen at −80°C.

### DNA extraction and pathogen screening

DNA was extracted from 176 ticks using a Qiagen DNeasy Tissue and Blood extraction kit. Prior to extraction, each tick was placed in liquid nitrogen and promptly crushed with a pestle. Conventional polymerase chain reaction (PCR) with a pan–genus primer pair was used to screen all ticks for both *Rickettsia* spp. and *Ehrlichia* spp. Primers and protocols for screening for *Rickettsia* spp. were chosen based on Kidd *et al*. (2008) and the research of Tucker (2017) (Table 1). The primers used for the screening of *Ehrlichia* spp. were developed by Doyle *et al*. (2005) and the protocols were based on the research of Tucker (2017). The specific instructions for the protocols relating to DNA extraction and PCR can be found in the Supplementary materials. For the screening of rickettsial pathogens, the primers 107F and 299R were used to produce a 209–212 bp amplicon 5′-hypervariable region of known ompA sequences (Kidd *et al*., 2008; Tucker, 2017). For the screening of *Ehrlichia* pathogens, the primers, DSB-321 and DSB-671 were used to amplify the 378 bp *Ehrlichia* dsb gene (Doyle *et al*., 2005). All samples were checked for amplification *via* gel electrophoresis (Supplementary materials). When testing for *Ehrlichia* spp., DNA samples were pooled, with ten ticks in each reaction. All positive samples were nano-dropped for quantitation and to test for purity, then sent to Genewiz (Gainesville, FL, USA) for Sanger sequencing. Sequences were aligned and analysed using Geneious Prime 2020.2.4 (https://www.geneious.com) and then blasted against NCBI Genbank.

**Table 1.** Primers used for pathogen screening of collected ticks

Pathogen	Primer	Direction	Amplicon (bp)	Targeted gene	Sequence
*Rickettsia* spp.	107F	Forward	209–212	*Rickettsia* ompA region	5′-GCT TTA TTC ACC ACC TCA AC-3′
	299R	Reverse			5′-TRA TCA CCA CCG TAA GTA AAT-3′
*Ehrlichia* spp.	DSB-321	Forward	378	*Ehrlichia* dsb gene	5′-TTG CAA AAT GAT GTC TGA AGA TAT GAA ACA-3′
	DSB-671	Reverse			5′-GCT GCT CCA CCA ATA AAT GTA TCY CCT A-3′

### Statistical analyses

To analyse a tick's likelihood to quest during the 10 min assay, a binary logistic regression in the program JMP (JMP Pro 15.0; 2019, SAS Institute) was used with habitat type, burn history, tick life stage (nymph/adult) and pathogen infection status as independent variables. To analyse average questing height and time spent questing, only ticks that quested during the 10 min assay were used (*n* = 73) using generalized linear models with identity link functions. Response variables were log 10 transformed to fit model assumption of normally distributed residuals, and both models included the same independent variables: habitat type, burn history, life stage and infection status. We originally included a habitat type × infection status interaction term, but it was not significant (*P* > 0.05), so it was removed for model simplification.

## Results

Out of 176 ticks collected in OSBS, 52 tested positive for *R. amblyommatis* [overall prevalence = 30%; 28% (26/94) in successional hardwood habitats, 32% (26/82) in xeric hammock habitats]. The homology for these samples with *R. amblyommatis* varied from 90.26 to 100%. None of the ticks collected tested positive for *Ehrlichia* infections. No evidence was found that habitat type, burn history, infection status or life stage altered the questing likelihood for ticks during the experimental assay (all *P* > 0.31; Table 2). Similarly, none of our independent variables appeared to have an effect on the average height at which ticks quested (all *P* > 0.31; Table 2).

**Table 2.** Summary of general linear mixed models predicting questing propensity, average questing height and total time spent questing

Independent variable	*df*	*χ*^2^-value	*P* value
Questing propensity			
Habitat type	1	0.17	0.68
Burn history	1	0.34	0.56
Life stage	1	1.03	0.31
Infection status	1	0.31	0.58
Questing height			
Habitat type	1	1.11	0.2919
Burn history	1	1.87	0.1716
Life stage	1	0.02	0.8811
Infection status	1	0.81	0.3683
Time spent questing			
Habitat type	1	6.99	0.008*
Burn history	1	2.65	0.10
Life stage	1	0.005	0.94
Infection status	1	3.85	0.05*

Significant effects are denoted with an asterisk.

**Fig. 2.**
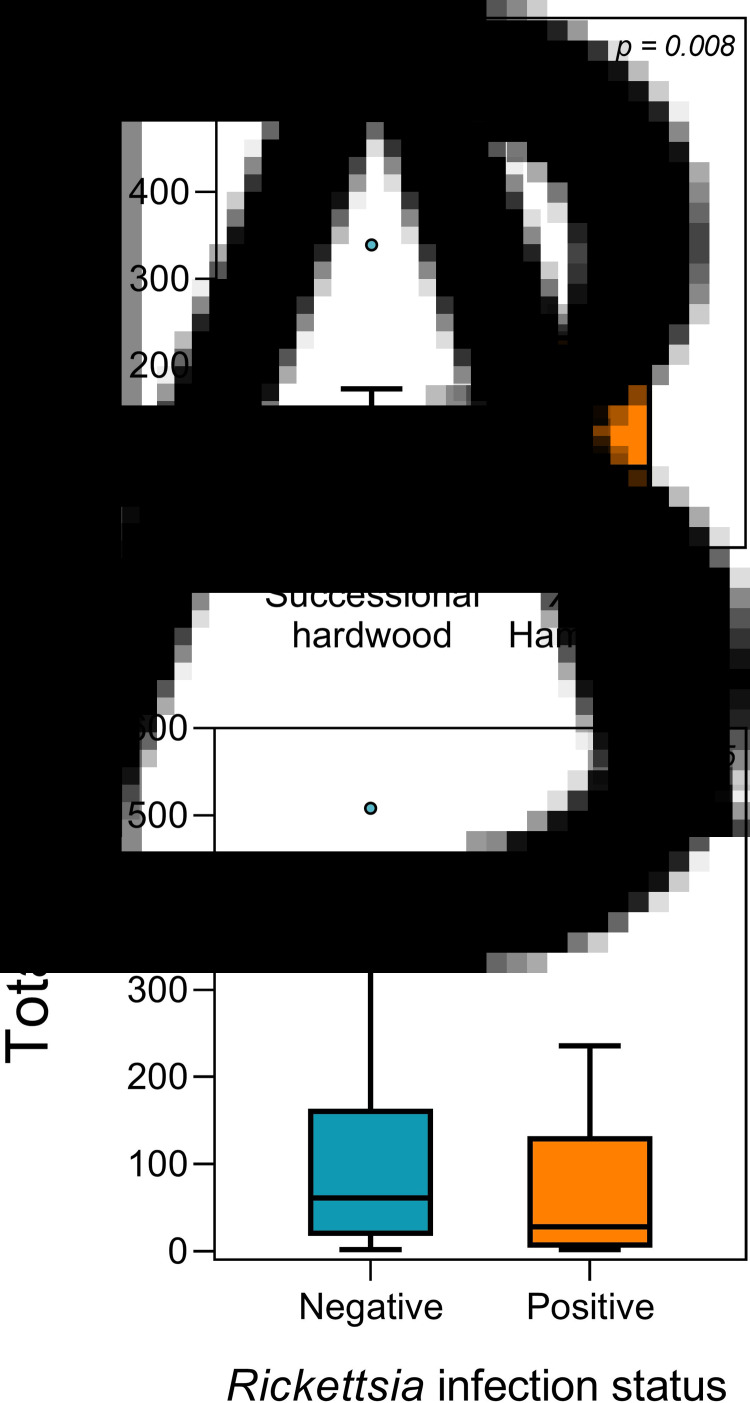
Time (s) spent questing by *Amblyomma americanum* in (A) two different habitats or (B) when infected or uninfected. Boxplots extend from the 25th to 75th percentiles, and the midline denotes the median. Whiskers generated using the Tukey method and outliers are shown outside of the whiskers.

Ticks collected from xeric hammock habitats spent over twice as long (151 s on average) engaging in questing behaviour compared to ticks collected from successional hardwood forests (58 s on average) (*df* = 1, *χ*^2^ = 6.99, *P* = 0.008; Table 2; Fig. 2A). Also, a weaker effect was detected where ticks that tested positive for *R. amblyommatis* infection spent less time questing compared to uninfected ticks, with infected ticks spending 85 s on average questing and uninfected ticks spending 112 s (*df* = 1, *χ*^2^ = 3.85, *P* = 0.05; Table 2; Fig. 2B). Tick-questing duration did not differ between adults and nymphs, nor did burn history appear to affect questing duration (all *P* > 0.10; Table 2).

## Discussion

A major factor in the prevention of tick-borne diseases is understanding how intrinsic and extrinsic factors influence tick host-seeking behaviour across different habitats. Here, we assessed how habitat type, burn history and infection status influenced host-seeking behaviours in *A. americanum*. We found infection prevalence with *R. amblyommatis* to be 30%, which corresponds to recent similar studies in Florida that found prevalence to be 29% (De Jesus *et al*., 2019). Although habitat, burn history and infection status did not affect the questing likelihood of a tick or the questing height, the amount of time a tick spent questing was significantly affected by the habitat type and infection status. We found that ticks collected from xeric hammock habitats spent twice as long questing compared to ticks collected from successional hardwood forests. We also found a weaker effect that ticks infected with *R. amblyommatis* spent less time questing compared to uninfected ticks.

Different habitat types possess a variety of environmental characteristics that can each influence a tick's host-seeking abilities. For example, a multitude of studies have shown the key roles that humidity and temperature play in tick host-seeking behaviours (Semtner and Hair, 1973; Schulze *et al*., 2001; James *et al*., 2013; Beck and Orozco, 2015), so ticks are more likely to be found in microhabitats that reduce desiccation risk. Here, we found that ticks collected from xeric hammocks spent twice as long questing as the ticks collected from successional hardwood forests. If ticks in different habitat types exhibit differences in questing behaviour within individual questing bouts (58 s *vs* 151 s during a 10 min observation period), this may generate important functional differences in questing behaviour across tick lifetimes. Interestingly, these differences in tick behaviour were displayed despite questing assays occurring in the same standardized location, rather than at the site of collection. Future studies should address how environmental characteristics and tick experience influence long-term physiological changes or behavioural tendencies that influence host-seeking behaviour. Reciprocal transplant studies between different habitats would be particularly helpful to address these questions. Although we measured similar levels of relative humidity, and temperature during our collection periods (Table S1), there are numerous differences in understory complexity. For example, the average wind speed in xeric hammock was higher than that of successional hardwood forests, most likely due to a more open understory (Fig. 1). We also noted that xeric hammocks had a much thicker layer of leaf litter, which can act as a protective barrier from stressors like high heat and low humidity (Semtner and Hair, 1973; Koch and Burg, 2006). The most notable difference between these habitats is the lack of structural complexity in xeric hammocks compared to successional hardwoods. In *I. scapularis*, immature tick densities appear to increase with the density of woody vegetation and decrease with the density of herbaceous vegetation (Adler and Spielman, 1992). It may be that ticks in habitats with fewer suitable substrates available for questing (i.e., less vegetation and course woody debris) will quest for longer durations once a suitable substrate is discovered. That is, each questing opportunity is more valuable when there are fewer opportunities to quest. Testing tick-questing behaviour before and after the removal/addition of vegetation to natural habitats could shed light on this phenomenon. Additionally, which hosts are present in each habitat type and the host's size could play a role in their host-seeking behaviours like questing heights (Portugal *et al*., 2020).

We found no effect of burn history on tick-questing behaviour. In both xeric hammocks and successional hardwoods, ticks were collected from sites that had been subjected to prescribed burns either within 4 years or those that had burned longer ago. Gleim *et al*. (2014) found that long-term prescribed burn histories reduced tick counts in surveys across northern Florida and southern Georgia (Gleim *et al*., 2014). We are unaware of any studies which have assayed tick-questing behaviour across habitats with varying burn histories, but perhaps the effects of prescribed burn latencies on the scale of 1–7 years is not great enough to alter tick host-seeking behaviour. Given that prescribed burns have widespread use in tick control programmes (Davidson *et al*., 1994; Gleim *et al*., 2014), more studies are needed to identify how burn history and habitat characteristics interact to influence tick host-seeking behaviours.

How vector behaviour changes when carrying human pathogens is a major focus of vector-borne disease studies. Much research has focused on the effects of infections on *Ixodes* tick behaviour, but far fewer studies have focused on *A. americanum* despite its increasing population densities and expanding range (Sagurova *et al*., 2019; Benelli, 2020). Here, we found that ticks infected with *R. amblyommatis* spent less time engaging in host-seeking behaviours compared to their uninfected counterparts. Romashchenko *et al*. (2012) found that *Borrelia*-infected *I. persulcatus* ticks quested at greater heights but were also significantly slower-moving than the uninfected ticks (Romashchenko *et al*., 2012). Interestingly, Lefcort and Durden (1996) found that *Borrelia*-infected *I. scapularis* adults were less active and quested at lower heights (Lefcort and Durden, 1996). Although both of these studies were looking at infections with *B. burgdorferi*, they were not looking at the same species of tick. This difference in species could have accounted for this difference in questing activity found. This change in activity levels in infected ticks also has been correlated with an increased resistance to heat stress (Alekseev and Dubinina, 2000; Busby *et al*., 2012; Herrmann and Gern, 2015). It is possible that infection with *R. amblyommatis* could have an effect on physiological aspects of *A. americanum* and this could lead to greater heat stress expression or greater risk of desiccation. Physiological impacts on the vector such as these could lead to differences in questing capabilities which impact transmission dynamics. Given that we collected ticks from the wild, assessed their behaviour and then sequenced them for pathogens, the risk of type-II error from small/uneven sample sizes is possible. Future studies could utilize artificial infections in lab-reared ticks to generate more even sample sizes between infected and uninfected ticks.

An important aspect of this research is understanding the relationship between disease risk and human recreational land use. The habitat types that ticks were collected from, xeric hammock, successional hardwood forest and abandoned fields/pastures were chosen with human land use in mind. Xeric hammock habitat types are commonly used as hiking trails, as they have open spaces that are easy to traverse (Friend, 2004). Interestingly, ticks from xeric hammocks may pose a greater risk of tick-borne diseases as ticks found in this area spent twice as long questing. Future studies could collect ticks on hiking trails cut through successional hardwood forests to test for differences in questing behaviour between trails and intact forests. Interestingly, although we originally sought to collect ticks from abandoned fields/pastures, these sites were removed from analyses due to the small number of ticks obtained from this habitat type. This is fortunate as this habitat type is often used for recreation (Plieninger *et al*., 2015). Given that *A. americanum* is an increasingly important disease vector in Florida and elsewhere, future studies should incorporate host-seeking behaviour into investigations of human disease risk across habitats when designing management plans for recreational spaces. Additionally, future research could perform quantitative PCR to determine if behavioural changes associated with infection in ticks are intensity dependent. As another caveat, having removed ticks from their original habitat and conducting the questing assay elsewhere exposes the ticks to novel environmental variables that could alter their questing behaviour. Comparing our results to studies where ticks are tested *in situ* would be helpful. Additionally, adding a transparent barrier between the assay chamber and the researcher could assist in preventing any breath/CO_2_ from the researcher onto the ticks which could encourage the ticks to quest.

This study provides a foundation for understanding the potential joint effects of extrinsic factors (habitat type and burn history) and intrinsic factors (pathogen infection) on tick host-seeking behaviour. Although studies on *R. amblyommatis* infection influencing tick traits are still developing, there is evidence that this infectious agent can potentially complicate RMSF diagnoses in human hosts (Barrett *et al*., 2014; Karpathy *et al*., 2016). Thus, it is important to understand the ways in which *R. amblyommatis* can affect a primary vector, *A. americanum*, so we can better understand how this pathogen is spread to humans, wildlife and domesticated animals. Future research should focus on the physiological basis of infection-induced changes to vector behaviour in the *A. americanum*–*R. amblyommatis* system. Additionally, this should be tested in ticks collected from a variety of habitat types so that we can better link vector behaviour with human disease risk across different habitat types.

## Supplementary material

For supplementary material accompanying this paper visit https://doi.org/10.1017/S0031182021001554. click here to view supplementary material
