# Supplementary material for: The effects of habitat type and pathogen infection on tick host-seeking behaviour
Source: Parasitology. 2021 Sep 6;149(1):59–64. doi: 10.1017/S0031182021001554 (PMC8862009; doi:10.1017/S0031182021001554)
Supplement: Supplementary file 1 [file S0031182021001554sup001.docx]

Supplemental Materials

**DNA Extraction**

The instructions of the Qiagen DNeasy Tissue and Blood extraction kit were followed with slight changes. This protocol was followed for each individual tick.

- A microcentrifuge tube with a single tick inside was dipped into liquid nitrogen for between 5-10 seconds and then the tube was immediately removed, open, and the tick was grinded with a pestle.
- Pipette 180 μL of ATL buffer was used to wash off the pestle into the microcentrifuge tube.
- Pipette 20μL proteinase K into the tube
- Vortex for approximately 15 seconds
- Put tube into hot water bath for 10 mins at 56°C
- Pipette 200μL of AL buffer into the tube
- Vortex for approximately 15 seconds
- Put tube into hot water bath for 10 mins at 56°C
- Pipette 200μL of cold ethanol into tube
- Vortex for approximately 15 seconds
- Move liquid from tube into filter tube
- Centrifuge at 8000 RPM for 1 minute
- Discard flow through
- Pipette in 500 AW1 wash buffer
- Centrifuge 8000 RPM 1 PM
- Discard flow through
- Pipette in 500 AW2 wash buffer
- Centrifuge 14000 RPM for 2 min (Dry Spin)
- Take filter top and put into new tube
- Pipette in 50μL warm elusion buffer
- Let sit 1 min
- Centrifuge 1 min 8000 RPM
- Discard filter
- Collect flow through containing DNA
- Keep in extracted DNA in freezer

**Conventional PCR**

***Rickettsia*** **spp.**: Each reaction had a total of 50μL, composed of: 25μL of mastermix, 3μL of forward primer (107F), 3μL of reverse primer (299R), 14μL of nuclease free water, and 5μL of DNA.

The PCR conditions inside the thermal cycler (Bio-Rad, Hercules, CA):

- One cycle of a denaturation step at 95°C for 2 mins
- 40 cycles of:
  - 95°C for 30 seconds
  - 56°C for 45 seconds
  - 72°C for 30 seconds
- One cycle of 72°C for 7 mins
- The machine is left at 4°C forever until ended.

***Ehrlichia*** **spp**.: Each reaction had a total of 45μL composed of: 25μL of mastermix, 2μL of forward primer (Dsb-321), 2μL of reverse primer (Dsb-671), 10μL of nuclease free water, and 6μL of DNA. The DNA used was pooled with 1μL from each tick with 10 ticks per reaction.

The PCR conditions inside the thermal cycler (Bio-Rad, Hercules, CA):

- One cycle of a denaturation step at 95°C for 5 mins
- 40 cycles of:
  - 95°C for 15 seconds
  - 60°C for 1 minute
  - 72°C for 1 minute
- One cycle of 72°C for 7 mins
- The machine is left at 4°C forever until ended.

**Gel Electrophoresis**

A 2% agarose gel was used and run at 120 volts for 40 minutes. 20μL of TrackIt™ 50 bp DNA Ladder (Thermofisher Scientific, Waltham, MA) and 6X DNA loading dye (Thermofisher Scientific, Waltham, MA) were used to determine the amplicon size and to better photograph the gel under the UV light. The ingredients for the gel were as follows:

- 6g agarose (Thermofisher Scientific, Waltham, MA)
- 30mL of 10x TAE buffer (Thermofisher Scientific, Waltham, MA)
- 270mL DI water)
- 30μL of Ethidium Bromide (VWR Life science, Radnor, PA)

**Table S1**: Data collected using a Kestrel® 3000 Wind Meter of environmental variables (temperature, relative humidity, and wind speed) at each habitat type.

|  | **Successional Hardwood Site 1**  **(B-1b)** | | **Successional Hardwood Site 2**  **(B3)** | |
| --- | --- | --- | --- | --- |
|  | Day 1 | Day 2 | Day 1 | Day 2 |
| **Temperature** | 30.83 | 31.1 | 32.4 | 32.3 |
| **Relative humidity** | 60.5% | 62.6% | 54.5% | 73.0% |
| **Average wind velocity** | 0.0 mph | 1.2 mph | 0.0 mph | 0.0 mph |
|  | **Xeric Hammock Site 1**  **(D-2a)** | | **Xeric Hammock Site 2**  **(C-8a)** | |
|  | Day 1 | Day 2 | Day 1 | Day 2 |
| **Temperature** | NA | 32.5 | NA | 32.9 |
| **Relative humidity** | NA | 65.9% | NA | 63.3% |
| **Average wind velocity** | NA | 2.0 mph | NA | 0.6 mph |
